# HPV genotype distribution among women with normal and abnormal cervical cytology presenting in a tertiary gynecology referral Clinic in Ethiopia

**DOI:** 10.1186/s13027-018-0201-x

**Published:** 2018-08-14

**Authors:** Dawit Wolday, Muluken Derese, Solomon Gebressellassie, Bekure Tsegaye, Wondwossen Ergete, Yirgu Gebrehiwot, Orit Caplan, Dana G. Wolf, Shlomo Maayan

**Affiliations:** 1 Medical Biotech Laboratory, Addis Ababa, Ethiopia; 2 Department of Medical Microbiology, Parasitology and Immunology, College of Health Sciences, Addis Ababa University, Addis Ababa, Ethiopia; 3 Department of Pathology, College of Health Sciences, Addis Ababa University, Addis Ababa, Ethiopia; 4 Department of Obstetrics & Gynaecology, College of Health Sciences, Addis Ababa University, Addis Ababa, Ethiopia; 5 The AIDS Center, Hadassah University Hospital - Hebrew University, Jerusalem, Israel; 6 Path Medical Services, Addis Ababa and Mekelle University College of Health Sciences, Mekelle, Ethiopia

**Keywords:** Cervical cancer, Cytology, HPV, Genotype distribution, Ethiopia, Sub-Sahara Africa

## Abstract

**Background:**

Cervical cancer is the second most prevalent cancer among women of child-bearing age in Ethiopia. The aim of this study was to determine human papilloma virus (HPV) genotype distribution among HIV-negative women with normal and abnormal cervical cytology results.

**Methods:**

We investigated a consecutive of 233 HIV-negative women between December 2008 and March 2009 presenting in a Tertiary Gynecology Referral Clinic in Ethiopia. Screening was done by Pap cytology and HPV detection and genotyping method was nested PCR (direct amplification with MY09/MY11 primers, followed by nested amplification with GP5/GP6 primers) and sequencing of the nested products. Sequencing of the non-purified nested PCR products was performed following re-amplification with Big dye terminator, using the GP6 primer.

**Results:**

Of the 233 study participants, 92 (39.5%) had abnormal cytology. All women with abnormal cervical cytology had positive HPV DNA compared to only 48.9% of those presenting with normal cytology. Of these women, the frequency of high-risk (HR)-HPV was 83.2% and its prevalence in women with abnormal cervical cytology was significantly higher than those with normal cytology (92.4% vs. 71%, *p* < 0.0001). The most frequent genotypes identified were HPV16 (44.1%), followed by HPV35 and HPV45 (each 6.2%), HPV31 (4.4%), HPV56 (3.7%), HPV18 and HPV59 (each 3.1%), HPV58 (2.5%) and HPV39 (1.9%). While the most common HR-HPV infections among women with normal cytology were HPV16 (20.3%), followed by HPV35 (8.7%), HPV56 and HPV58 (each 5.8%), HPV18, HPV31 and HPV39 (each 4.4%), HPV45 (2.9%) and HPV59 and HPV68 (each 1.5%), the most common HR-HPV infections in women with abnormal cytology included HPV16 (62%), followed by HPV45 (8.7%), HPV 31, HPV35 and HPV59 (each 4.4%), and HPV18, HPV52 and HPV56 (each 2.2%). We also noted low prevalence of multiple HPV infections in women with normal or abnormal cytology. Multivariable logistic analysis showed that residing in rural area (OR 3.24, 95% CI: 1.13–9.30), being multipara (OR 7.35, 95% CI: 1.78–30.38) and having abnormal cervical cytology results (OR 6.75, 95% CI: 1.78–25.57) were all independently associated with HPV16 genotype.

**Conclusions:**

Our study revealed a significant risk of infection with HR-HPV, in particular with HPV16 genotype, in women attending a referral center in Ethiopian women presenting with or without abnormal cervical cytology. Moreover, Pap smear cytology missed a significant proportion of women compared to those who were identified by PCR for HR-HPV infections. In addition, the PCR method we used was not suitable for sensitive detection of co-existent multiple infections. Data from the present study indicate that currently available HPV vaccines could prevent nearly 67% of all cervical cancer cases in women in Ethiopia.

**Electronic supplementary material:**

The online version of this article (10.1186/s13027-018-0201-x) contains supplementary material, which is available to authorized users.

## Background

Cervical cancer is the second most common cancer among women worldwide and the leading cause of mortality in developing countries [1]. It is the second most prevalent cancer among women of child-bearing age in Ethiopia [2, 3]. The country has a population of 29 million women aged 15 years and older who are at risk of developing cervical cancer [2]. Few epidemiological investigations undertaken in Ethiopia have documented that more than 90% of invasive cervical cancers are attributed to infection with oncogenic HPV; HPV type 16 has been identified to be the most frequent genotype accounting for 56% to 91% of the cases of cervical cancers occurring in Ethiopia [4–9]. Although the prevalence of high-risk HPV genotypes has been documented in those who develop overt cervical cancer in the country, there is paucity of data on the rate of HPV infection or genotype distribution among women with normal cervical cytology or those with abnormal cervical lesions. Furthermore, differences in sensitivity and specificity of the HPV detection and genotyping methods (e.g., hybrid capture, direct PCR, nested PCR, line arrays, sequencing) and combinations of them could potentially result in variations of the HPV prevalence and genotypes found in previous studies of African and Ethiopian women [4–9]. A detailed understanding of the molecular epidemiology of HPV diversity among women presenting with diverse cervical cytological status is warranted before consideration is given in the future use of available HPV-based vaccines. Hence, we undertook this study with the aim to determine the HPV infection and genotype distribution among women with normal and abnormal cervical cytology results.

## Methods

### Study participants

Study participants were recruited between December 2008 and March 2009 from the population of women attending the out-patient clinic of the Department of Gynecology and Obstetrics, Tikur-Anbessa Tertiary General Hospital, Health Sciences College, Addis Ababa University, Addis Ababa, Ethiopia. The hospital is a tertiary referral facility with primary responsibility in medical education. Thus, several patients are referred to this hospital, which is located in the capital city of Ethiopia, for advanced diagnosis, management and care. The out-patient clinic of the Department of Gynecology and Obstetrics has several units, of which this specific study was undertaken at the Oncology Unit of the department, which runs once per week. On average, about 1500 patients are seen annually in the Unit. At presentation, potential participants were informed about the study. The criteria for enrollment included: age above 18 years, willingness to participate in the study and willingness for HIV counseling and testing. Exclusion criteria were age less than 18 years, pregnancy, women on menstrual cycle, women who did not start sexual activity, presence of cervical cancer and unwillingness to participate in the study.

### Data collection

All women who agreed to participate in the study provided written informed consent. At the enrolment visit, data on detailed socio-demographic information, as well as a clinical history and information on behavioral risk factors were collected using standardized questionnaire by senior nursing staff. Trainings on sensitive data management topics were provided to all staff involved in the research project, which included topics like data recording, data collection, data validation, double entry, correction of data, data export, data security and safety, harmonisation and merging datasets. Common data safety and quality assurance policy were applied. In addition, full clinical examination was done by senior gynecologist and two cervical specimens were collected: one was for smear onto glass slides to be used for routine cytology and a second cervical swab was taken for HPV detection. The latter was placed in 1 ml of RNA/DNA stabilization reagent and then immediately placed in ice before transfer where it was stored at -80 °C until further analysis for HPV DNA PCR and genotyping at the Virology Laboratory, Hadassah University Hospital in Jerusalem, Israel. In addition, whole blood specimen was collected for HIV-1 serology.

### Laboratory investigations

#### Cervical cytology

The smears were stained with the Pap stain and routinely reported by a pathologist according to the criteria of the Bethesda classification system [10]. Diagnosis was attempted only on smears with adequate number of cells. All smears were reviewed independently by two senior pathologists who were unaware of the clinical or other laboratory findings to avoid bias. In the event of discrepant smear result readings, both pathologists reviewed the slides together and consensus was reached on final diagnosis and grading.

#### HPV detection and typing

Cervical swabs were put onto lysis buffer and were incubated at 37 °C for 2–3 h and then heated to 96 °C for 10 min. The samples were subjected to DNA extraction (Roche – Magnapure). Sample adequacy for DNA amplification was validated by examination of the cellular gene RNase P, which was assayed using the Taqman RNase P kit (Applied Biosystems) according to the manufacturer’s instructions. Five μL of the eluted DNA were subjected to nested PCR amplification using primers derived from HPV L1 region (MY09/MY011 consensus primers for the first amplification, followed by a second amplification reaction, using primers GP5/GP6, as described [11]). The size of the nested PCR products was 150 base pairs and the nested PCR products were not purified. The nested PCR products were re-amplified using the GP6 primer with Big dye terminator (Applied Biosystems) followed by sequencing (3730 XL DNA analyzer; Applied Biosystems). Following amplification, 10 μL of PCR products were electrophoresed through 2% agarose gel and were stained using ethidium bromide and examined under UV transillumination for expected amplicons. The HPV genotypes were assigned to the resulting sequences using The Basic Local Alignment Search Tool (BLAST; NCBI - NIH). The sequences obtained corresponded to one HPV (unless a mixed infection was specifically identified, as stated). HPV genotypes 16, 18, 31, 33, 35, 39, 45, 51, 52, 56, 58, 59 and 68 were considered as high risk (HR)-HPV and other HPV genotypes, including 26, 53, 66, 67, 70, 73, and 82 were classified as “possible/probable” (pHR) carcinogens according to the recent review of the International Agency for Research on Cancer (IARC) assessing carcinogenicity of biological agents [12].

#### Serological testing for HIV

HIV testing was performed based on National Algorithm for HCT. Briefly, HIV screening was done by KHB test kit (Shanghai Kehua Bioengineering Co. Ltd., China) and reactive samples were tested again by HIV 1/2 STAT-PAK assay (Chembio Diagnostic Systems Inc., USA). Discordant samples were subjected to a tie-breaker test using Unigold HIV (Trinity Biotech Plc., Ireland).

### Ethical considerations

The protocol was reviewed and approved by Institutional Review Committees of Addis Ababa University Health Sciences College and Hadassah University Hospital as well as the National Ethical Review Committee of the National Science and Technology Ministry, Addis Ababa, Ethiopia.

### Statistical analysis

Questionnaire responses, cervical cytology results, HPV results and other laboratory data were entered onto a database. Through-out the study, double-data entry was undertaken in order to assure quality of the data. Proportions were compared using pearson’s *X*^2^ test and student’s *t*-test. Logistic regression analysis was also used to identify the association between the presence of HPV infection and a range of demographic, behavioral and morbidity characteristics. Multiple logistic regression analysis was carried out in order to see the effects of confounding factors. *P* value of less than 0.05 was considered indicative of statistical significance. All statistical analyses were performed using the Stata (version 13, Stata Corporation, College Station, Texas, USA) and SPSS (SPSS Inc., Chicago, IL, USA) Statistical Software packages.

## Results

### Demographic characteristics

A total of 233 HIV-negative women were enrolled for the study. The mean (95% CI) age of the patients was 41.7 years (40.1–43.2). Furthermore, a significant proportion of these women had multiple sexual partners, were multipara with low income status and no formal education (Additional file 1: Table S1). As noted in above, the methodology section, the Oncology Unit of the Department of Gynecology and Obstetrics where the study was undertaken is responsible for investigating referral cases. In the study group, thus, 34.8% were from rural area and 44.2% were referral cases. Those coming from rural area also being referred represented 33.5% of all the patients seen.

### Cytology results

Of the total of 233 patients included and evaluated for cytology examination, the results revealed that 141 (60.5%) samples were normal while 92 (39.5%) were abnormal. Of those with abnormal cytology results, the prevalence of high grade squamous intraepithelial lesion (HSIL) at 68.5% (95% CI: 58.8–78.2) was significantly higher than low grade squamous intraepithelial lesion (LSIL) at 6.5% (95% CI: 1.4–11.7) and invasive cervical carcinoma (ICC) at 25% (95% CI: 16–34.1). The prevalence of abnormal cytology findings among women also presenting with STI appears to be significantly higher from those without STI (75.7%, 95 CI: 67.3–84.1 vs. 10.8%, 95% CI: 5.4–16.2, *p* < 0.0001). Furthermore, of those women referred, only 21.3% (95%CI:14.4-28.1) had normal cytology results that was significantly lower compared to those with abnormal cytology (79.3%, 95% CI:70.9-87.8, *p* < 0.0001).

### HPV prevalence and genotypes in relation to cervical cytology status

**Fig. 1:**
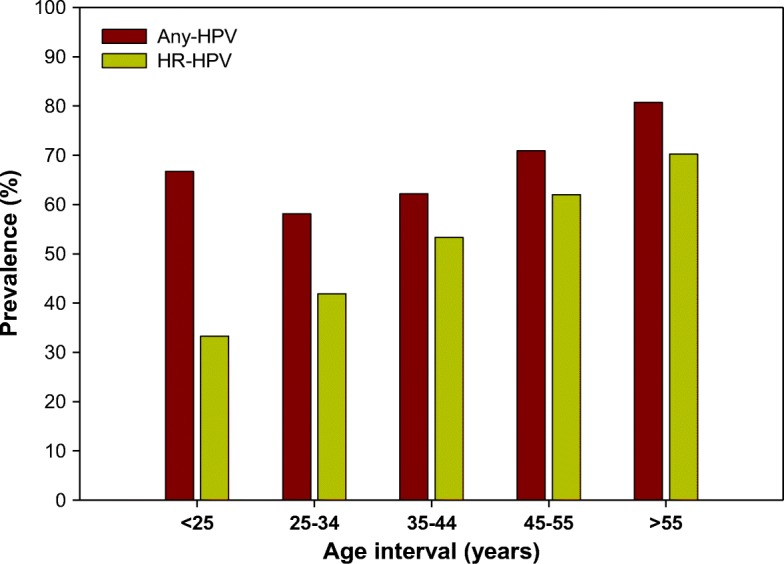
Age-specific prevalence of human papilloma virus (HPV) DNA positive (any-HPV) and high-risk (HR)-HPV

Figure 1 shows age-specific prevalence of any-HPV DNA positive and HR-HPV DNA positive women. It appears that the prevalence for HPV, in particular high-risk (HR)-HPV genotypes, increased steadily with increasing age.

Table 1 shows the HPV prevalence and HR-HPV genotype distribution in relation to cytology status. The prevalence for any HPV in the study population was 69.1%. However, further analysis revealed that women with abnormal cervical cytology had significantly higher HPV prevalence than those presenting with normal cytology (100%, 95% CI: 92.7–100.0 vs. 48.9%, 95% CI: 40.6–57.3, *p* < 0.0001). The overall prevalence for HR-HPV was 83.2%. The prevalence of HR-HPV among women with normal cervical cytology was significantly lower than those with abnormal cytology (71%, 95%CI: 60–82 vs. 92.4%, 95% CI: 86.7-97.9; *p* < 0.0001). HPV was identified in all specimens from patients with abnormal cytology results and the prevalence of HR-HPV was 83.3%, 95.2% and 87% among those with LSIL, HSIL and ICC, respectively.

**Table 1:** HPV prevalence and genotype distribution in relation to cervical cytology status

	All cases (*N* = 233)	Normal cytology (*N* = 141)	Abnormal cytology (*N* = 92)			
			TOTAL (*n* = 92)	LSIL (*n* = 6)	HSIL (*n* = 63)	ICC (*n* = 23)
Any HPV	161 (69.1)	69 (48.9)	92 (100)	6 (100)	63 (100)	23 (100)
LR-HPV	24 (15.2)	17 (25.8)	7 (7.6)	1 (16.7)	3 (4.8)	3 (13)
Multiple HPV	3 (1.86)	1 (1.5)	2 (2.2)		2 (3.2)	
Untypable	3 (1.9)	3 (4.4)				
HR-HPV	134 (83.2)	49 (71.0)	85 (92.4)	5 (83.3)	65 (95.2)	20 (87.0)
HPV-16	71 (44.1)	14 (20.3)	57 (62.0)	4 (66.7)	40 (63.5)	13 (56.5)
HPV-18	5 (3.1)	3 (4.4)	2 (2.2)		2 (3.2)	
HPV-31	7 (4.4)	3 (4.4)	4 (4.4)		4 (6.4)	
HPV-35	10 (6.2)	6 (8.7)	4 (4.4)		3 (4.8)	1 (4.4)
HPV-39	3 (1.9)	3 (4.4)				
HPV-45	10 (6.2)	2 (2.9)	8 (8.7)	1 (16.7)	4 (6.4)	3 (13.0)
HPV-52	2 (1.2)		2 (2.2)			2 (8.7)
HPV-56	6 (3.7)	4 (5.8)	2 (2.2)		1 (1.6)	1 (4.4)
HPV-58	4 (2.5)	4 (5.8)				
HPV-59	5 (3.1)	1 (1.5)	4 (4.4)		4 (6.4)	
HPV-68	1 (0.6)	1 (1.5)				
pHR-HPV						
HPV-53	2 (1.2)	2 (2.9)				
HPV-66	1 (0.6)	1 (1.5)				
HPV-70	4 (2.5)	3 (4.4)	1 (1.1)		1 (1.6)	
HPV-73	3 (1.9)	2 (2.9)	1 (1.1)		1 (1.6)	

*LR* low-risk, *HR* high-risk, *pHR* probable-HR, *LSIL* low-grade squamous intraepithelial lesion, *HSIL* high-grade squamous intraepithelial lesion, *ICC* invasive cervical carcinoma

Of all HR-HPV infections (*n* = 134), the most frequent genotypes was HPV16 (44.1%), followed by HPV35 and HPV45 (each 6.2%), HPV31 (4.4%), HPV56 (3.7%), HPV18 and HPV59 (each 3.1%), HPV58 (2.5%), HPV39 (1.9%) and HPV52 (1.2%). While the most common HR-HPV infections among women with normal cytology were HPV16 (20.3%), followed by HPV35 (8.7%), HPV56 and HPV58 (each 5.8%), HPV18, HPV31 and HPV39 (each 4.4%), HPV45 (2.9%) and HPV59 and HPV68 (each 1.5%), the most common HR-HPV infections in women with abnormal cytology included HPV16 (62%), followed by HPV45 (8.7%), HPV 31, HPV35 and HPV59 (each 4.4%), and HPV18, HPV52 and HPV56 (each 2.2%).

Overall, the prevalence of HPV16 is significantly higher among women with abnormal cytology compared to the prevalence among women with normal cytology (62%, 95% CI: 51.8–72.1 vs. 20.3%, 95% CI: 10.6–30, *p* < 0.0001). The most frequent HR-HPV types in LSIL lesions were HPV16 (66.7%) and HPV45 (16.7%); in HSIL lesions were HPV16 (63.5%), HPV31, HPV45 and HPV59 (each 6.4%), HPV35 (4.8%), HPV18 (3.2%) and HPV56 (1.6%), and in ICC lesions were HPV16 (56.5%), HPV45 (13%), HPV52 (8.7%) and HPV35 and HPV56 (each 4.4%).

### Risk factors associated with HR-HPV and HPV16 infections

Table 2 shows risk factors associated with HR-HPV infections. The most important risk factors associated were lack of formal education, being referred for treatment of gynecological disorder, women from rural area, being multipara, women presenting with sexually-transmitted infections (STI) and those with abnormal cytology results. However, age, marital status, low income, having multiple sexual partners and using oral contraceptive were not associated with HR-HPV infection. Multiple logistic regression analysis demonstrated that residing in rural area was the only factor independently associated with HR-HPV infection.

**Table 2:** Risk factors associated with HR-HPV positivity

Characteristics	HR-HPV positivity		Univariable		Multivariable	
	N/Total	Prevalence (%)	OR	95% CI	AOR	95% CI
Age > 55	20/134	14.9	2.19	0.48–9.99		
Single marital Status	72/134	53.7	1.08	0.47–2.47		
No formal education	91/134	67.9	6.05	2.38–15.39	3.28	0.92–11.72
Referral cases	83/134	61.9	4.65	1.84–4.28	0.45	0.08–32.70
Rural	62/134	46.3	6.89	1.89–4.76	8.01	1.32–48.67
Low income	122/134	91.0	0.81	0.17–3.86		
Multiple sexual partners	56/134	41.8	1.22	0.52–2.86		
Multiparity	103/128	80.5	2.58	1.04–6.35	1.09	0.39–3.04
Any STI	83/134	61.9	3.25	1.36–7.79	1.00	0.26–3.89
Oral contraceptive use	2/134	1.5	1.06	0.79–1.43		
Abnormal cytology	85/134	63.4	4.96	1.96–4.12	2.43	0.44–13.53

*OR* = odds ratio, *AOR* = adjusted odds ratio; *STI* = sexually transmitted infections

In this study, HPV16 was the most prevalent of all HR-HPV genotypes. Thus, we further assessed determinant factors associated with HPV16 infections (Table 3). The most important risk factors were lack of formal education, being referred for treatment of gynecological disorder, women from rural area, earning low income, being multipara, women presenting with STI and those with abnormal cytology results. However, multiple logistic regression analysis demonstrated that residing in rural area, being multipara and having abnormal cervical cytology results were independently associated with HPV16 infection.

**Table 3:** Risk factors associated with HPV16 positivity

Characteristics	HPV16 positivity	Univariable	Multivariable			
	N/Total	Prevalence (%)	OR	95% CI	AOR	95% CI
Age > 55	12/71	16.9	1.63	0.66–4.02		
Single marital Status	37/71	52.1	0.91	0.49–1.70		
No formal education	59/71	83.1	6.43	3.04–13.58	2.31	0.85–6.28
Referral cases	51/71	71.8	3.33	1.72–6.48	0.64	0.18–2.24
Rural	40/71	56.3	3.35	1.74–6.48	3.24	1.13–9.30
Low income	69/71	97.2	5.31	1.15–24.55	1.22	0.21–6.96
Multiple sexual partners	21/71	29.6	0.42	0.21–0.81		
Multiparity	65/68	95.6	12.83	3.73–44.25	7.35	1.78–30.38
Any STI	50/71	70.4	2.72	1.41–5.25	0.28	0.07–1.03
Oral contraceptive use	1/71	1.4	1.04	0.85–1.27		
Abnormal cytology	57/71	80.3	6.40	3.11–13.17	6.75	1.78–25.57

*OR* = odds ratio, *AOR* = adjusted odds ratio; *STI* = sexually transmitted infections

## Discussion

In this current study, we identified a significant proportion of women with abnormal cytology results (39.5%, 95%CI: 33.2–45.8). This is somewhat higher than a previous reported results done in Ethiopia showing a prevalence of precancerous lesions of 22.1% [13], but is consistent with other studies from Congo that reported a prevalence of 36.1% [14]. Recently conducted systematic review by Ogembo et al. reported great variations in HPV prevalence among women with normal cytology across African countries [15]. Specifically, the Southern African region had the highest prevalence (57.3%), followed by Eastern Africa (42.2%), Western Africa (27.8%) and Northern Africa (12.8%). Another report from rural Ethiopia reported overall prevalence of HPV at 17.3% [8]. The overall prevalence of HR-HPV was also significant in the current study (49/141 = 34.8%) among the women we studied with normal cervical cytology results. Though our study results indicate that the HPV prevalence among women with normal cytology in Ethiopia is consistent with reports for Eastern Africa [15], our findings appear much higher than previously reported for Ethiopia [8] and Western Africa [16]. Nevertheless, cytological evaluation was not performed in the Ethiopian study suggesting differences in prevalence. The findings from our study showing the increasing prevalence of any HPV infection with cervical disease grade concurs with the recent review findings of Ogembo et al. for several African countries [15].

Our study also revealed a significant risk of infection with HR-HPV. Moreover, the overall prevalence of HR-HPV and the most prevalent genotype, HPV16, were also significant among women with normal cervical cytology (Table 1), indicating that Pap smear cytology missed a significant proportion of women compared to those who were identified by PCR methods with HR-HPV and those with HPV16 infections. Our findings are in agreement with analysis results showing low sensitivity of Pap smear compared to HPV DNA testing [15–19]. Overall, Pap smear misses 24.7% women who were identified by PCR as HR-HPV infections and potentially preventable by currently available vaccines in Ethiopia. Indeed, 37.8% of the women identified as HR-HPV infections by PCR occurring in Pap smear-negative women that we studied have genotypes that are potentially preventable by currently available 9-valent HPV vaccines.

The prevalence of HR-HPV found in our study was 83.2% of any HPV DNA positive cases. Moreover, the prevalence of HR-HPV ranged from 71% among women with normal cytology to 95.2% in women with HSIL. In our patients, the prevalence of HR-HPV in women with ICC was found to be 87% (95% CI: 72.07–101.85), which is in accordance with the 93% reported by Abate E et al. from Ethiopia [7]. The most frequent HR-HPV genotype in the current study was HPV16 accounting for prevalence of 44.1% in all HPV DNA positive subjects, and prevalence of 20.3% and 62% among women with normal and abnormal cervical cytology conditions, respectively. Previous studies from Ethiopia have revealed prevalence of HPV16 ranging from 56 to 91% in women presenting with high-grade cervical lesions [4–9]. The differences in sensitivity and specificity of the HPV detection and genotyping methods (e.g., hybrid capture, direct PCR, nested PCR, line arrays, sequencing) and combinations of them could potentially result in variations of the HPV prevalence and genotypes found in previous studies of African and Ethiopian women. Overall, HPV16 was the most predominant genotype from HPV DNA positive samples and was also the most prevalent in all cytological categories. Other common HR-HPV types were HPV35, HPV45, HPV18 and HPV31 with a combined prevalence of 20%.

In the current study, the most important independent risk factors associated with HR-HPV was related to residence in rural setting. Though underlying conditions have not been studied in detail in the current study, it might be related to lower socio-economic status, as noted in previous studies from Mexico [20], differences in health-seeking behaviors or distance to health facilities. Independent risk factors related to HPV16 infections, the most frequent genotype noted, were residence in rural setting, being multipara and presenting with abnormal cervical cytology. Similar findings were reported from previous studies conducted in Ethiopia [8] and other African countries [14, 21].

### Strengths and limitations of the study

This study, unlike previous studies done in Ethiopia, included evaluation of cytological status. Indeed, this is the first study to investigate HPV prevalence and genotype distribution among women with normal cytology. The data provided herewith provide some information about the background HPV genotype distribution and also the lower diagnostic sensitivity of Pap smear compared to HR-HPV DNA PCR methods in identifying risk factors for cervical cancer [15–19].

The limitation of the study includes the inclusion of a significant proportion of women who were referred for advanced diagnosis and management and those from rural area resulting in increased prevalence rates of abnormal cytology results as well as HPV infections. The fact that the selected women are mostly referred because of gynecological problems might limit the use of the current data for epidemiological purposes. The HPV genotype distribution in the current study was performed almost 10 years ago, nonetheless data from Ethiopia are still hardly available [2] and we believe that the publication of our findings will serve, at least in part, fill this gap.

## Conclusions

To our knowledge, this study is the first comprehensive assessment of the overall prevalence and distribution of HPV genotypes among Ethiopian women presenting with normal and abnormal cervical cytology findings. Data from the present study indicate that vaccines currently available could prevent nearly 67% of all cervical cancer cases in women in Ethiopia. Moreover, our data suggests that newly licensed 9-valent HPV vaccines would prevent almost 80% of cancer cases in Ethiopian women. Current WHO guidelines advocate “screen and treat” approach for resource constrained settings [22]. Such programs have been implemented in several countries in sub-Saharan Africa, including in Ethiopia [23]. Nonetheless, our results, along with the findings that only 10% of the women screened based on Visual Inspection with Acetic acid were identified for treatment [23] suggest the need for further evaluation of the usefulness of the “screen and treat” approach in Ethiopia.

Ethiopia has no comprehensive national cancer control program. Likewise, there is no national HPV vaccination program. A pilot feasibility study is underway in two sites of the country before scale-up for national HPV vaccination programme. A more comprehensive and representative molecular epidemiology studies are also warranted to understand the real situation in Ethiopia.

## Additional file

Additional file 1: **Table S1.** Socio-demographic characteristics. (DOCX 15 kb)

## Abbreviations

CI: Confidence interval
HPV: Human papillomavirus
HR: High risk
HSIL: High-grade squamous intraepithelial lesion
ICC: Invasive cervical cancer
LR: Low risk
LSIL: Low-grade squamous intraepithelial lesion
