# Supplementary material for: HPV genotype distribution among women with normal and abnormal cervical cytology presenting in a tertiary gynecology referral Clinic in Ethiopia
Source: Infect Agent Cancer. 2018 Aug 14;13:28. doi: 10.1186/s13027-018-0201-x (PMC6092870; doi:10.1186/s13027-018-0201-x)
Supplement: Supplementary file 1 — Table S1. Socio-demographic characteristics. (DOCX 15 kb) [file 13027_2018_201_MOESM1_ESM.docx]

**Additional file 1. Socio-demographic characteristics**

| **Characteristics** | **(*N*=233)**  **Prevalence (%)** |
| --- | --- |
| **Age (mean, 95% CI)** | 41.7 (40.1-43.2) |
| **Age interval (mean, 95% CI)** |  |
| < 25 | 3.8 (1.37-6.36) |
| 25-34 | 18.5 (13.44-23.47) |
| 35-44 | 19.3 (14.21-24.42) |
| 45-54 | 33.9 (27.78-40.01) |
| > 55 | 24.5 (18.90-30.02) |
| **Single marital Status** | 57.1 (50.68-63.48) |
| **No formal education** | 53.2 (46.76-59.67) |
| **Referred** | 44.2 (37.78-50.63) |
| **Rural** | 34.8 (28.60-40.92) |
| **Low income** | 87.6 (83.28-91.82) |
| **Multiple sexual partners** | 39.1 (32.74-45.37) |
| **Multiparity** | 73.6 (67.60-79.57) |
| **Contraceptive use** | 0.4 (0.00-1.27) |
| **STI** | 44.2 (37.78-50.63) |

STI = Sexually Transmitted Infections
